# Low Carbohydrate Diets for Diabetic Cardiomyopathy: A Hypothesis

**DOI:** 10.3389/fnut.2022.865489

**Published:** 2022-04-20

**Authors:** Sabine Kleissl-Muir, Bodil Rasmussen, Alice Owen, Caryn Zinn, Andrea Driscoll

**Affiliations:** ^1^School of Nursing and Midwifery, Deakin University, Geelong, VIC, Australia; ^2^Centre for Quality and Patient Safety, School of Nursing and Midwifery, Institute for Health Transformation, Deakin University, Geelong, VIC, Australia; ^3^The Centre for Quality and Patient Safety, Institute of Health Transformation -Western Health Partnership, Western Health, St Albans, VIC, Australia; ^4^Faculty of Health and Medical Sciences, University of Copenhagen, Copenhagen, Denmark; ^5^Faculty of Health Sciences, University of Southern Denmark and Steno Diabetes Centre, Odense, Denmark; ^6^School of Public Health and Preventive Medicine, Monash University, Melbourne, VIC, Australia; ^7^Human Potential Centre, Faculty of Health and Environmental Sciences, Auckland University of Technology, Auckland, New Zealand; ^8^Department of Cardiology, Austin Health, Heidelberg, VIC, Australia

**Keywords:** diabetic cardiomyopathy, low carbohydrate diet, ketogenic diet, heart failure, insulin resistance, diabetes

## Abstract

Elevated blood glucose levels, insulin resistance (IR), hyperinsulinemia and dyslipidemia the key aspects of type 2 diabetes mellitus (T2DM), contribute to the development of a certain form of cardiomyopathy. This cardiomyopathy, also known as diabetic cardiomyopathy (DMCM), typically occurs in the absence of overt coronary artery disease (CAD), hypertension or valvular disease. DMCM encompasses a variety of pathophysiological processes impacting the myocardium, hence increasing the risk for heart failure (HF) and significantly worsening outcomes in this population. Low fat (LF), calorie-restricted diets have been suggested as the preferred eating pattern for patients with HF. However, LF diets are naturally higher in carbohydrates (CHO). We argue that in an insulin resistant state, such as in DMCM, LF diets may worsen glycaemic control and promote further insulin resistance (IR), contributing to a physiological and functional decline in DMCM. We postulate that CHO restriction targeting hyperinsulinemia may be able to improve tissue and systemic IR. In recent years low carbohydrate diets (LC) including ketogenic diets (KD), have emerged as a safe and effective tool for the management of various clinical conditions such as T2DM and other metabolic disorders. CHO restriction achieves sustained glycaemic control, lower insulin levels and successfully reverses IR. In addition to this, its pleiotropic effects may present a metabolic stress defense and facilitate improvement to cardiac function in patients with HF. We therefore hypothesize that patients who adopt a LC diet may require less medications and experience improvements in HF-related symptom burden.

## Introduction

### Diabetes and Heart Failure

Having reached pandemic levels globally, the number of people living with diabetes is forecast to exceed 700 million by 2045 (1). T2DM is the most common form of diabetes, and accounts for almost 90% of registered cases (1). T2DM typically develops in adulthood, however due to changes in lifestyle and diet, its prevalence is increasing in young adults and children (1). Although diabetes monitoring and screening strategies are in place, it is estimated that 1 in 2 diabetes cases stay undiagnosed (1). Equally, HF has been identified as an epidemic, with an estimated 38 million cases worldwide (2). Despite the current standard of care, a systematic review analyzing the cost of HF in the USA between 2014 and 2020 estimated that HF accounts for 108 billion dollars per year (3), owing to prolonged hospitalisations and increased readmission rates frequently described in the context of the disease.

Vascular complications, specifically stroke and myocardial infarction, are responsible for many premature deaths in people living with diabetes, however HF is now the leading cause of death in the adult-onset diabetes mellitus population (4, 5).

Close to 30% of patients living with T2DM concurrently exhibit HF, and up to 40% of HF patients also present with T2DM (6). This strong link exists independently of traditional risk factors such as age, smoking or CAD (7). Hyperglycaemia and poor blood glucose control are strongly associated with HF.

Literature suggests, each 1% increase in mean glycaemic levels as referenced by glycosylatedhaemoglobin (HbA1c), is associated with an 8% increased risk for HF. IR appears to be an independent predictor of HF and is diagnosed in up to 70% of HF patients, in the absence of common risk factors including diabetes itself (8–10).

Historically, HF in the context of diabetes (DMCM), was first described in 1972. Rubler et al. (11) discovered changes consistent with HF during autopsies on four diabetic patients. These changes were not attributable to CAD, hypertension or valvular disease. Further research has established diabetes as independent risk factor for HF and acknowledges DMCM as a distinct entity (12). To date, there is no universally recognized definition for DMCM which poses a challenge in determining global or country specific incidence and prevalence (12).

## Pathophysiological Aspects of DMCM

The pathophysiology of DMCM is complex and has been previously well-described (12, 13). For the purpose of this paper, we will provide a brief general description and focus on the pathophysiological aspects relevant for our hypothesis.

## Systemic and Cardiac IR

Our review into the pathogenesis of DMCM as well as HF has highlighted the importance of systemic IR and impaired myocardial insulin signaling. Prognostically, diabetes drastically increases adverse outcomes in HF, however literature describes IR as the main driver associated with myocardial impairment (14). IR may already be present in youth and precede categorical diabetes by decades, hence going undetected in otherwise healthy individuals, posing a significant independent health risk (15). A recent study by Banerjee et al. (9) investigated the risk of incident HF associated with IR in a sample of 4,425 participants from the Cardiovascular Health Study undertaken in the USA. The authors found that fasting insulin level, a surrogate for IR, was an independent predictor of HF even in the absence of traditional risk factors including T2DM (9).

IR is defined as a physiological abnormality through which insulin-mediated glucose uptake and disposal into muscle and adipose tissue becomes impaired. Consequently, more insulin needs to be excreted to overcome the defect in insulin action and to maintain blood glucose homeostasis (15, 16).

The resulting compensatory hyperinsulinemia and IR are strongly linked to T2DM, cardio-renal metabolic syndrome [including dyslipidaemia, inflammation, coagulopathy, hypertension, non-alcoholic fatty liver disease (NAFLD)] and other conditions such as cancer and neurological disorders (17). Systemic IR plays a crucial role in the pathogenesis of DMCM causing systemic metabolic perturbations which in turn cause impairment of insulin signaling and cardiac IR (17, 18).

Cardiac IR may develop independent of systemic factors contributing to systemic IR such as obesity and overnutrition, increase reactive oxygen species (ROS), neurohormonal and/or cytokine activation and the development of cardiac IR (17, 19).

Normal physiological conditions require a high-performing heart, necessitating the generation of 5 kg of adenosine triphosphate (ATP) per day utilizing predominantly free fatty acids (FFA) and to a lesser extent glucose, small amounts of ketones and amino acids (20). This metabolic flexibility is essential for an efficiently pumping heart which is able to adapt to any workload (20). In the stressed heart, the preferred substrate is glucose as it provides a more efficient fuel therefore reducing myocardial oxygen demand. Alterations in insulin signaling fundamentally change this metabolic flexibility (21).

Hyperglycaemia, IR and hyperinsulinemia are the driving forces across all stages of DMCM (22). In a state of overnutrition, excess CHO, especially high-dose fructose is not cleared by the small intestine and so enters the portal system and hepatocytes. There it increases the production of fatty acids and storage of fat in the liver *via de novo* lipogenesis (23). High levels of circulating fatty acids exacerbate fat storage in the liver and manifest as atherogenic dyslipidaemia characterized by elevated triglyceride levels, a decrease in high-density lipoprotein (HDL) levels and changes in the size of low-density lipoprotein (LDL) particles (24). Moreover, the increased circulation of FFA interrupts insulin signaling causing cardiac IR, fatty acid deposition in cardiac tissue and a shift in substrate metabolism (25). Toxic by-products of glucose accumulate in patients with hyperglycaemia causing the formation of advanced glycation end products (AGE) and ROS. These harmful compounds cause oxidative stress and adversely affect cellular function in the cardiac tissue leading to cardiac stiffness and diastolic dysfunction, hence directly contributing to DMCM (12).

This persistent inflammatory state not only leads to peripheral nerve damage but also contributes to autonomic denervation of the heart known as cardiac autonomic neuropathy (CAN) (26). CAN has been associated with tachycardia, decreased exercise tolerance, cardiac arrhythmias and sudden cardiac death (26). In the IR state, where sodium retention and volume excess are already present, the maladaptive physiological changes described above lead to an inappropriate activation of the Renin-Angiotensin-Aldosterone System (RAAS). Activation of RAAS further compounds the effect of diastolic dysfunction seen in DMCM. Together, the above alterations associated with high blood glucose and hyperinsulinemia provide a substrate for DMCM as well as ischaemic cardiac events (27).

## The Clinical Picture of DMCM

The existing literature suggests four main stages of DMCM (Table 1). In stage 1 DMCM, also named the “Early Stage” (28), the clinical symptoms of HF such as dyspnoea, decreased exercise tolerance and pedal oedema may be absent or vague. Hyperglycaemia and impaired insulin signaling promote systemic inflammation and activation of the sympathetic nervous system (18, 21). Echocardiological examinations reveal evidence of early left ventricular (LV) hypertrophy and diastolic cardiac dysfunction. These early pathological changes may be present in over 60% of asymptomatic (pre)diabetic patients (29). At this stage systolic function is still preserved, referred to as HF with preserved ejection fraction (HFpEF) (12, 18, 21, 28–30).

**Table 1:** Stages of DMCM.

**Classification**	**Stage 1 DMCM (early stage)**	**Stage 2 DMCM (middle stage)**	**Stage 3 DMCM (middle- late stage)**	**Stage 4 DMCM (late stage)**
Symptoms of heart failure/dyspnoea	NYHA I (asymptomatic)	NYHA II	NYHA II - III	NYHA II - IV
Function (echo)	Diastolic dysfunction (HFpEF) normal systolic function	Diastolic and systolic dysfunction (HFpEF and HFrEF), EF <50%, low flow	Worsening diastolic and systolic dysfunction (HFpEF and HFrEF), EF <50%, low flow	Worsening diastolic and systolic dysfunction (HFpEF and HFrEF), EF <50%, low flow
Morphology	Increased LV mass (hypertrophy)	Increased LV mass (hypertrophy), dilatation, fibrosis	Dilatation, fibrosis, microangiopathy	Dilatation, fibrosis, micro and macro angiopathy (CAD)
Cellular mechanisms	Cardiac steatosis with increased FFA, shift of substrate metabolism	AGE formation, IR, cell death (apoptosis), necrosis, fibrosis, mild CAN, increased RAAS	Features from early and middle stages + hypertension and microvascular changes, severe CAN	Features from early and middle stages + hypertension and microvascular changes, severe CAN and CAD
Metabolism	Prediabetes, metabolic syndrome	Early diabetes, hyperglycaemia	Overt diabetes, IR	Overt diabetes, IR
Biomarkers of diabetes	Hyperglycaemia and elevated HbA1C	Hyperglycaemia and elevated HbA1C	Hyperglycaemia and elevated HbA1C	Hyperglycaemia and elevated HbA1C

*CAD, coronary artery disease; NYHA, New York Heart Association functional classification of HF; EF, ejection fraction; CAN, cardiovascular autonomic neuropathy; HFpEF, HF with preserved ejection fraction; HFrEF, HF with reduced ejection fraction*.

In stages 2 and 3 DMCM (Middle and Middle/Late Stage respectively), patient symptomatology worsens, and systolic function slowly declines. The RAAS is activated and the high levels of FFA damage cardiac myocytes causing progressive contractile dysfunction (28). Disruption of the autonomic cardiac nervous system becomes apparent further worsening hypertension and HF symptoms (29). In stage 4 DMCM (Late Stage), the condition may progress to severe HF with reduced ejection fraction (HFrEF) and the clinical manifestations of HF become evident (29–31). In addition to this, both micro- and macroangiopathy may now be promoting acute coronary events and sudden cardiac death (28, 29). Due to the frequent hospitalisations and functional decline experienced during later stages of DMCM, patients face many challenges which not only impact their life but the lives of their family (32).

## Pharmacological Management of DMCM

The cardiac dysfunction seen in DMCM is particularly difficult to treat and until recently pharmacological therapies have been based on HF management. Gene-based therapy is predicted to play an important role in targeting both diabetes and HF in the future, however substantial research is needed to explore these therapies in clinical practice (31). Recent HF guidelines focus on the pharmacological treatment of hyperglycaemia and cardiac risk factor modification (6, 33).

Sodium-glucose co-transporter type 2 inhibitors (SGLT2i) show benefits in the management of DMCM, suggesting decreased mortality and significantly less hospitalisations specifically in patients with diabetes and HFrEF (31, 34). SGLT2i exert their properties by stopping the reabsorption of glucose in the kidney. By removing excess glucose with the help of these agents, multiple clinical benefits become apparent (35, 36). SGLT2i are effective in lowering blood glucose levels and have a diuretic effect, with patients excreting an additional 500 mls/day often necessitating the adjustment of diuretics. In addition to this, research also proposes weight and blood pressure reduction in people with diabetes taking the medication (13, 35).

Recently incretin-based drugs such as glucagon-like peptite-1 (GLP-1) analogs have emerged as a treatment option for diabetic patients (21, 37). Incretin hormones in healthy humans aid in the control of blood glucose levels by augmenting insulin excretion while simultaneously regulating glucagon release from the pancreas (38, 39). In patients with T2DM this “incretin effect” is diminished, therefore incretin mimetics may improve blood glucose levels, satiety and outcomes in these individuals (39).

## Current Dietary Management of DMCM

There are currently no dietary recommendations specific to the DMCM population. Nutritional strategies for the prevention and treatment of diabetes and HF have been proposed, focusing mostly on LF calorie-restricted diets as well as dietary salt reduction. As such the available guidelines from the American and European Heart Associations currently recommend a “heart healthy diet” low in saturated fat and emphasizing the intake of whole grains, fruits, legumes, vegetables, and nuts. Small amounts of lean white meat and fish are encouraged (40, 41).

The most endorsed LF dietary pattern is the Dietary Approaches to Stop Hypertension (DASH) diet (40). This mostly plant-based diet has an added focus on lowering dietary sodium intake (<2.3 g of sodium/day). Previous hallmark trials, such as the DASH trial and the DASH-Sodium trial have demonstrated the diet's beneficial effects on cardiac biomarkers, specifically elevated blood pressure (42). A subsequent secondary analysis of stored specimens from the DASH-Sodium trial revealed improved biomarkers of cardiac injury and strain but not on inflammation (42).

In the most recent systematic review and meta-analysis of randomized controlled trials (RCT) reporting on the effects of the DASH diet on systolic and diastolic blood pressure undertaken by Siervo et al. (43), the authors reported the DASH diet to be associated with a significant reduction in systolic and diastolic blood pressure, total cholesterol and LDL concentrations. However, other cardiometabolic markers such as blood glucose, HDL or triglyceride levels remained unchanged (43).

## The LC Diet

Dietary interventions are generally considered LC if the ingested dietary CHO falls below 130 g per day (measured in absolute amounts). This amount equates to <26% of daily calories coming from CHO (44). However, depending on the intended therapeutic use (e.g., epilepsy treatment, weight loss) the amount of CHO ingested per day may vary considerably and be as low as 20 g of net CHO per day (45). Table 2 depicts the current Clinical Guidelines for Therapeutic Carbohydrate Restriction definitions for levels of CHO restriction (44).

**Table 2:** Clinical Guidelines for Therapeutic Carbohydrate Restriction definitions of LC diet, published with permission from Hite et al. (44).

⇒ VLCK (very low-carbohydrate ketogenic) diets recommend 30 g or less of dietary carbohydrate per day (46). Deliberate restriction of kilocalories (kcals) is not typically recommended. ⇒ LCK (low-carbohydrate ketogenic) diets recommend 30–50 g of dietary carbohydrate per day (47). Deliberate restriction of kcals is not typically recommended. ⇒ RC (reduced-carbohydrate) diets recommend 50–130 g of dietary carbohydrate per day, a level that is higher than levels listed above and lower than the U.S. DRI for carbohydrate. Deliberate restriction of kcals may or may not be recommended at this level. ⇒ MC-CR (moderate-carbohydrate, calorie-restricted) diets recommend more than 130 g of dietary carbohydrate per day with a range of 45–65% of daily kcals coming from carbohydrate. In most cases, kcals are also restricted to maintain energy balance or to achieve a deficit for weight loss. This dietary intervention reflects the amount of dietary carbohydrate typically found in “carbohydrate counting” interventions given to many people with type 2 diabetes. ^*^Definitions of carbohydrate levels depicted above may either refer to total CHO content or to non-fiber (net) grams of CHO, as fiber is not typically metabolized to glucos.

Definitions * are based on protocols currently in use or on definitions found in the literature.

Before the development of exogenous insulin, LC diets were considered the gold standard of treatment for diabetes. In recent years LC and KD have been rediscovered as safe and effective tools for the management of T2DM and other pathological conditions associated with IR such as polycystic ovary syndrome and some cancers. In addition to this, clinical research describes improvements in blood lipid profiles and blood pressure in diabetic patients following a LCKD (48–50). Lastly, recent meta-analysis of RCTs found LC diets to be superior for weight loss compared with other (LF) diets (51, 52).

LC diets strongly limit the consumption of grains, grain products, confectionary, sugar sweetened beverages, and trans fats. Small daily amounts of legumes, starchy vegetables and fruit may be incorporated in less restricted LC diets (44, 53). A well-formulated LC diet typically includes a variety of non-starchy, above ground and green leafy vegetables. To include sources of natural plant fats, the consumption of nuts, seeds, avocado, and olives are encouraged. Other food sources of natural fats such as butter and coconut are allowed.

To meet the recommended daily protein intake of 1.2–1.7 g/kg/estimated lean body mass, LC diet also include liberal consumption of animal proteins and unsweetened dairy products (44).

When dietary CHO is limited, the liver produces ketone bodies such as beta-hydroxybutyrate (BHB) and acetoacetate metabolized from fatty acids. These small, water soluble ketone bodies are able to provide an alternative fuel substrate for the heart, skeletal muscles and brain as they can cross the blood-brain barrier (54). As the amount of ketones rise in the blood, insulin secretion and blood glucose stabilize at reduced levels (55, 56). Lowered insulin levels and the resulting rise in glucagon in the blood mediate fat breakdown in adipose tissue and the liver, freeing more FFAs for ketogenesis (55).

## Hypothesis

Considering the evidence from peer reviewed literature, we hypothesize that a LC diet can ameliorate systemic IR and improve whole body metabolic and tissue function, which will subsequently positively impact cardiac function and the symptoms of DMCM. Specifically, we describe the impact of the LC diet on several metabolic pathways and its proposed flow-on effect on the heart.

## Controlling Postprandial Lipid and Glucose Dysmetabolism (Glucolipotoxicity)

Once ingested, dietary CHO is the only macronutrient directly broken down into glucose. As such CHO has the biggest impact on postprandial blood glucose levels and insulin secretion (57). On the other hand, protein and fat have far less direct effect on insulin levels (58).

High CHO foods and added sugars may significantly contribute to postprandial hyperglycaemia in patients with glucose metabolism disorders such as DMCM. This hyperglycaemic state may last for up to 4 h post meal. The ensuing toxic effects of hyperglycaemia (glucotoxicity and lipotoxicity) are not only a causative factors in the development of HF (59), but are observed to be an independent risk factor for cardiovascular disease, even more dangerous than elevated fasting blood glucose levels (60, 61). As the typical daily eating pattern generally consists of multiple meals, this toxic environment may persist all day (62).

In non-diabetic insulin sensitive individuals, the ROS generated under physiological conditions are counteracted by powerful intracellular antioxidant systems. In the diabetic heart an imbalance exists between ROS production and quenching, which leads to increased ROS exposure (62). Hyperglycaemia-mediated ROS are associated with cell death, abnormal cardiac remodeling and dysfunction in DMCM models (18, 63, 64). Further, hyperglycaemia increases the production of AGE and glucose flux into cardiac myocytes which further contributes to myocardial fibrosis and dysfunction (18, 63).

One of the biggest contributing factors to of hyperglycaemia is insulin sensitivity, as the elevated insulin and associated blood glucose levels in response to a CHO rich meal are only seen in individuals with IR (65). As both the quality and amount of CHO in the meal determines these postprandial peaks (58), omitting high CHO foods from the diet can not only normalize (postprandial) blood glucose levels, but also reverse IR (53, 59, 65).

Studies on the effects of LC diets on glycaemic control in T2DM are well-established. Sainsbury et al. (66) conducted a systematic review and meta-analysis assessing the effect of dietary CHO restriction on glycaemic control in adults with diabetes. LC diets were found to be superior for short term (3–6 months) reduction in HbA1c (66). This result was supported by the most recent systematic review and meta-analysis of published and unpublished randomized trial data, evaluating the efficacy and safety of LC and very LC diets for T2DM remission undertaken by Goldenberg et al. (67). The authors concluded that among the included 23 clinical trials comparing a LC (<130 g CHO/day) to a LF diet, LC diets achieved greater improvements in IR (determined by HOMA-IR) and higher T2DM remission rates after 6 months (remission was classified as HbA1c <6.5% and cessation of diabetes medication) (67). Whilst both systematic reviews determined that the dietary benefits diminished after 1 year (66, 67) an ongoing long term trial by Mckenzie et al. (68) yielded some promising results. In this open-label, non-randomized clinical trial, 349 patients with T2DM self-selected to either a remote continuous care intervention (*n* = 262) (LC <30 g CHO/day) or usual care (*n* = 87).

Diabetes-related outcomes were assessed after 1 and 2 years demonstrating significantly reduced HbA1c levels, weight and medication use. Following this, intervention participants were invited to take part in a 3 (and 5) year extension. The outcomes of 143 consenting participants were assessed at 3.5 years. Statistically significant improvements from baseline were recorded in HbA1c (−0.6 ± 0.1 266 from 7.4 ± 0.1%; *p* = 1.9 × 10^−5^) and the weight of participants (−10.9 ± 1.1 from 117.4 kg; *p* = 6.9 × 10^−17^).

Almost 72% of diabetes medication (excluding Metformin) were ceased. Most significantly, 22% of participants achieved diabetes remission (68).

In addition to glucotoxicity, postprandial energy metabolism in IR disorders such as DMCM is negatively affected in another way. In the IR skeletal muscles, the metabolic conversion of glucose (ingested CHO) to glycogen is markedly impaired. Consequently, the ingested CHO is diverted away from muscle glycogen synthesis and converted into fat *via* hepatic *de novo* lipogenesis, promoting atherogenic dyslipidaemia and NAFLD. Even in the absence of overnutrition, CHO are the major drivers of dyslipidaemia (69, 70).

A study by Petersen et al. (70) investigated the association between IR and the development of atherogenic dyslipidaemia in a cohort of lean, young, and otherwise healthy individuals with IR, compared to insulin sensitive, matched controls. Both groups were exposed to two high CHO meals. The authors found that whilst postprandial blood glucose levels were similar in both groups, *de novo* triglyceride lipogenesis [very low-density lipoprotein (VLDL)] production was increased more than 2-fold in IR participants (15.7 ± 1.5%; intergroup significance *p* = 0.00005) whilst HDL levels decreased by 20% (70). For this reason IR is thought to be one of the most powerful risk factors for heart disease (71).

Whilst traditional hypotheses state that LF diets lower LDL cholesterol, it may not be the quantity of LDL particles in the blood that is detrimental to the heart, but LDL quality (72). A diet high in sugar, and/or abnormalities in CHO metabolism may cause a significant derangement in serum lipid profile and damage to LDL particles (23). These glycated small, dense LDL particles (sdLDL) are prone to oxidative damage and easily penetrate the arterial wall, inducing inflammation and the formation of atherogenic plaques (72). In DMCM the cardiac steatosis and inflammation due to constantly elevated FFA levels are the hallmark of the disease (29, 73). This suggests lowering triglyceride levels may have therapeutic effect (73).

Apprehension toward the LC diet has been based mostly on traditional hypothesis which state that heart disease increases in direct proportion with LDL cholesterol levels, a consequence of increased fatty acid intake (24). However, new research has found no association with dietary saturated fat intake, cholesterol levels and cardiovascular disease (CVD) (24). A detailed systematic review and meta-analysis was recently undertaken by Kang et al. (74). The authors analyzed data from 14 prospective health studies involving 598,435 participants and stroke events. Kang et al. (74) found an inverse relationship between saturated fat intake and stroke risk, contributing to the evidence against dietary saturated fat restriction. Further, in a prospective population-based cohort study of 12.8 million healthy adults, Yi et al. (75) found that low total cholesterol levels were consistent with higher mortality rates, especially in people over 40 years of age (75). Most significantly, a 20 year longitudinal study enrolling 334 HF patients found low LDL levels to be consistent with worse prognosis in HF patients, especially when treated with statins (76). The authors suggest LDL to have an important anti-inflammatory role in HF which has an impact on improving outcomes (76). LC diets can be effective to improve elevated triglycerides, sdLDL and low HDL levels which are components of an atherogenic lipid profile (77).

In addition to the effects on HbA1c, the ongoing remote care intervention by Mckenzie et al. (68) has observed marked improvements of triglycerides and HDL levels in diabetic patients who have followed a LC diet for 3.5 years. A lipid subfraction analysis undertaken in the 262 study participants found that patients on the LC/KD had lower levels of sdLDL particles (type IIIb), increased HDL levels and unchanged levels in the atherogenic Apolipoprotein B (ApoB) and LDL levels, supporting the evidence that longer term LC diets may not adversely affect heart disease risk (49).

## Modifying Ketone Metabolism in HF

Ketosis (blood ketone range of 0.5–3mmol/L) is a metabolic state, critical to human survival and evolution (44, 78, 79). In early hunting and gathering cultures, where humans spent around 15 h of the day fasted and only small amounts of digestible CHO were consumed, the KD was the predominant dietary pattern (78). The modern human, exposed to a seemingly endless food supply is rarely fasted outside of sleep time, therefore hardly ever takes advantage of this metabolic flexibility.

Unlike the endocrine emergency diabetic ketoacidosis (DKA), nutritional ketosis is not associated with a shift in blood pH and is generally achieved if dietary CHO is restricted to <50 g of total CHO per day (79).

In the failing heart (DMCM) where the capacity to oxidize FAA and glucose is increasingly lost, ketones may provide an alternative fuel substrate and have favorable haemodynamic effects (80). Further, ketone bodies have anti-inflammatory properties and may be able to regulate the overactive sympathetic drive seen in HF (80).

A different approach to raise plasma ketone levels is the administration of exogenous ketones such as ketone esters (KE) or salts (20). Whilst research regarding ketone supplementation for HF is in its infancy, some authors suggest increased plasma ketone levels may have therapeutic potential in both HF and DMCM (20). In the first randomized crossover trial undertaken on humans with stable, chronic HF with HFrEF receiving a ketone salt infusion, Nielsen et al. (81) demonstrated a 40% increase in cardiac output with a subsequent 8% increase in left ventricular ejection fraction (LVEF).

Further, a decrease in both systemic and pulmonary vascular resistance was documented (81). This effect was observed, not only in the HFrEF group, but also the control group when receiving a ketone salt infusion and occurred without affecting myocardial energy efficiency. Of particular interest is that plasma ketone levels in the study were described to be in the physiological range, as often observed in people who consume a LC diet (81). In a different trial by Monzo et al. (82), a small sample of HFrEF patients was selected to either receive 25 g of oral KE solution or no intervention. In both groups venous and arterial blood samples were taken and several myocardial substrates such as oxygen, lactate, BHB, FFA, and glucose examined (82). The authors concluded that ketones may provide a more efficient and cardioprotective fuel in a failing heart, therefore enhancing cardiac performance (82).

Providing additional insight regarding the effects of LC diets on HF was a study recently undertaken by McCommis et al. (83). The authors demonstrated that mitochondrial pyruvate carrier 1 and 2 (MCP1, MCP2), enzymes gatekeeping pyruvate entry into mitochondria, are downregulated in both the failing human and rodent heart (83). This mishandling of pyruvate may contribute to the deprived energy state and pathological remodeling in the failing heart. Six-week-old cardiac specific MPC2 knockout mice (CS-MPC2-/-) were fed either a LCKD, a LF, or other control diet for 11 weeks.

The cardiac dysfunction and enlargement in the CS- MPC2-/- mice could be completely reversed with a LCKD diet. The scientists suggest that the improvements seen in the LCKD fed HF mice were not linked to the increase of circulating ketone bodies but rather the enhanced fatty acid oxidation associated with a LCKD. It was further observed that even diets with moderate CHO content which were non-ketogenic yielded similar results. Interestingly, ~35% of the MPC2-/- mice fed a LF diet died before reaching 17 weeks of age, the surviving mice displayed severe cardiac dysfunction and pulmonary oedema (83).

## LC Diet Outcome Closely Resembles SGLT2i Therapy

Under physiological conditions virtually all glucose is reabsorbed from the glomerular filtrate in the proximal tubule of the nephron. Three membrane proteins, namely Sodium Glucose Transporter (SGLT) 1, SGLT2 and Glucose Transporter 2 (GLUT2) are responsible for this to occur. In diabetes, where plasma glucose concentrations may exceed the re-absorptive capability of 220 mg/dl, glucose appears in the urine (84). The elevated insulin levels seen in T2DM and IR augment the expression of SGLT2 and so worsen not only hyperglycaemia but also sodium and fluid retention (85). It is estimated that in diabetes renal sodium reabsorption is increased to 25%, from 5% in the healthy state (86). Hypertension is a central feature of IR and as such part of the syndrome that is DMCM. In 1933, Chasis et al. (87) demonstrated that the above mechanisms could be reversed with a plant glycoside called phlorizin. After decades of research into this compound and the mechanisms of renal glucose transport, SGLT2i are now the preferred treatment for patients living with diabetes to reduce HF and CVD progression (41, 88). Whilst the beneficial effect of SGLT2i in improving all-cause mortality and hospitalization in HF patients is undisputed, authors caution that patients taking SGLT2i require ongoing monitoring for genital infections (89), euglycemic diabetic ketoacidosis (EDKA) (90), and adverse effects on bone health (91, 92).

**Figure 1:**
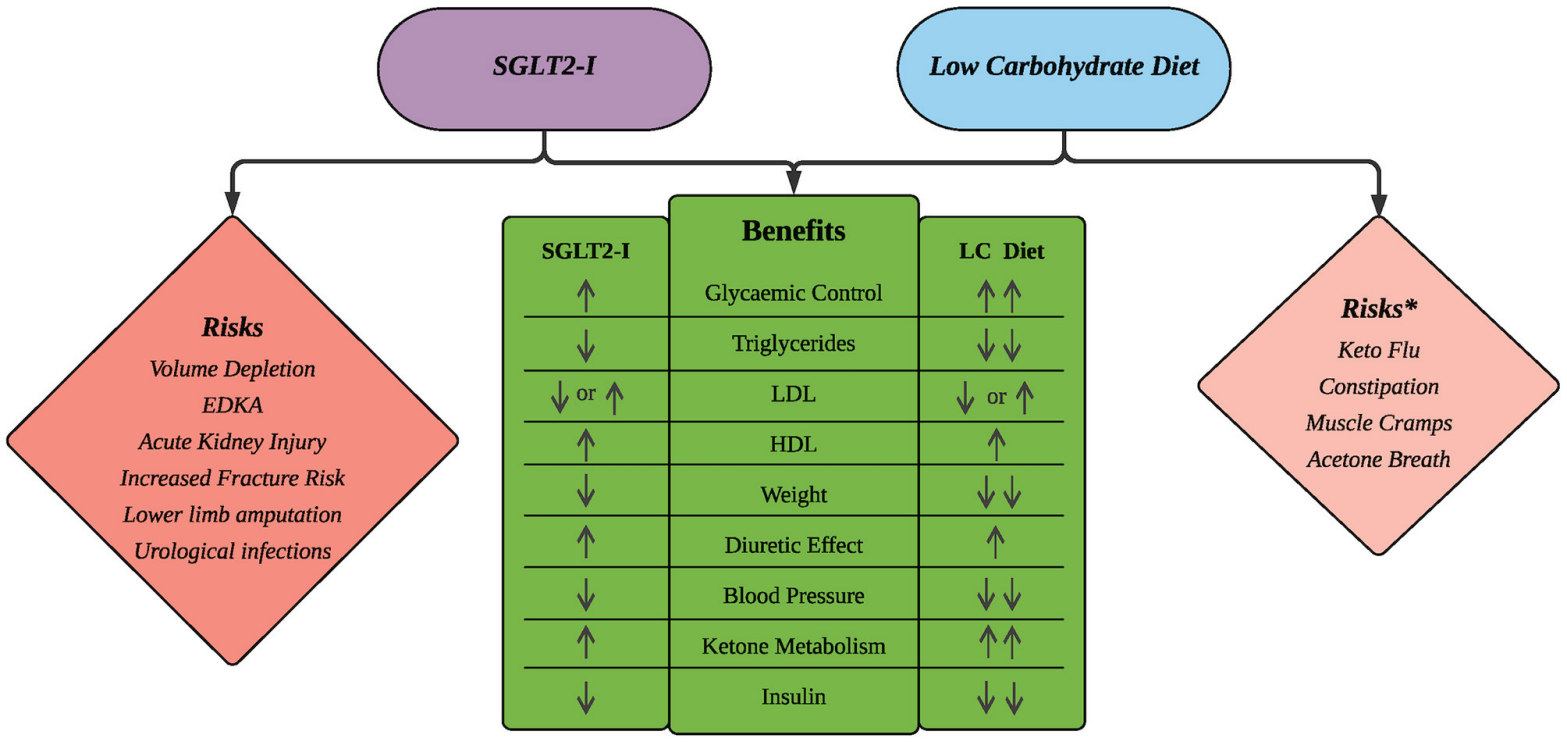
Comparison of benefits and risks of SGLT2i and LC diets. *Risks associated with the LC Diet are typically mild in severity and transient in nature. Number of arrows reflects magnitude of the intervention's effect.

Clinically proven benefits of SGLT2i such as weight loss, improved lipid profiles and blood pressure reduction are analogous to those of the LC diet (45, 50, 79, 93, 94) (Figure 1). This raises the question that, if the SGLT2i's foremost beneficial mode of action is to inhibit glucose reabsorption, could this alternatively be achieved by avoiding the consumption of CHO and added sugar? CHO restriction reduces hyperinsulinemia and postprandial hyperglycaemia, naturally increasing diuresis and sodium excretion by reducing SGLT2 expression in the kidney.

Analyzing clinical data from a GP practice in North England, Unwin et al. (50) describe a vast reduction in systolic blood pressure in 128 patients with T2DM who followed a LC diet for 2 years. Specifically, the authors observed an average 10.9 mmHg systolic blood pressure drop [interquartile range (IQR): 0.0, 22.0; *p* < 0.0001] and a decrease in diastolic blood pressure by 6.3 mmHg from baseline (IQR: 0.0, 12.8; *p* < 0.0001). This occurred despite 20% of participants having their antihypertensive medication ceased (50). Additionally, it should be noted that there was a 15% improvement in mean total cholesterol: HDL ratio (*p* < 0.0001). These findings demonstrate that the effects of a LC diet may be analogous to effects attributed to SGLT2i.

## Improving Body Composition

Obesity has been associated with the development of HF and is present in up to 50% of HF patients (2). A survival advantage, also known as the “obesity paradox” has been described for obese [grade 1 obesity, body mass index (BMI) of 25–29.9 kg/m^2^ (95)] patients living with HF, however this prognostic advantage has not been observed in patients with diabetes (33). Studies investigating the obesity paradox in HF often use BMI as a single measure of obesity, not taking into account body composition and other contributors to increased body weight. Powerful markers of cardiometabolic health such as increased visceral fat, dyslipidaemia, IR, hypertension, and NAFLD exist in both obese and normal weight individuals, therefore risk stratification should not be based on weight alone (95, 96).

Whilst an increased BMI may be beneficial for HF patients, weight reduction is known to improve overall metabolic health and increase cardiorespiratory fitness, leading to substantial improvements in disease outcomes (2). González Islas et al. (97) randomized 88 stable HF patients to either a LC diet or a low fat diet as recommended by the American Heart Association. The authors observed a statistically significant improvement in oxygen saturation (SaO_2_). These changes to SaO_2_ were only observed in the LC group (97).

Recent meta-analyses of RCTs have found LC diets to be safe and superior for both short and long term weight loss compared with LF diets (51, 52). In LC diets, associated weight loss may be facilitated *via* appetite suppression by circulating ketone bodies, lower levels of appetite controlling hormones such as leptin and ghrelin, and an increased intake of dietary protein and fat (93, 98). Further, LC diets improve the insulin to glucagon ratio, thus increasing lipolysis and favoring weight loss despite the often higher energy intake (93). LF high CHO diets, even if calorie- restricted, will not yield these effects as this metabolic advantage is uniquely ascribed to LC diets (93, 99).

Whilst LC diets can successfully reduce body fat, research shows that muscle mass and strength remain preserved. Body composition changes were evaluated by Gomez-Arbelaez et al. (100) in 20 obese patients following a very LC diet for 16 weeks. After 4 months participants had lost an average of 20 kg fat mass, whilst pre-intervention muscle mass was maintained (100). As sarcopenia and cachexia are associated with adverse outcomes in HF patients (101), the LC diet may offer an alternative approach to HF patients who would also benefit from weight loss.

Cardiac cachexia, defined by unintentional non-oedematous weight loss of >5% over a period of at least 6 months, poses a particular challenge in the management of HF patients. In the metabolically healthy human, insulin protects muscle mass by suppressing muscle proteolysis. In the IR state, such as in DMCM, where the anabolic effect of insulin is diminished, skeletal muscle decreases, and sarcopenia ensues (102–104). Further, the increased proteolysis and upregulation of neurohumoral pathways lead to a catabolic state, provoking skeletal muscle loss and tissue wasting, which is typical in patients with cardiac cachexia (105). To date, studies concerning LC/LCKD and cachexia have been mainly undertaken in animal cancer models. These studies have demonstrated a reduction in tumor growth and reduced weight and muscle loss (106).

It remains to be elucidated if a well-balanced LC diet as part of a multimodal intervention could lessen the anabolic resistance and so attenuate cardiac cachexia in HF patients.

## Evidence From Human Trials

To date, evidence from human RCTs concerning LC diets for the dietary management of DMCM are scarce. Von Bibra et al. (107) describe a significant effect of a LC diet compared to a LF diet on the diastolic cardiac function in their RCT. Two matched groups of 16 T2DM patients without heart disease (52 ± 7 years, BMI 34 ± 6 kg/m^2^) were observed in a parallel and partial cross over design during a 3-week rehabilitation program. Whilst both the LC and the LF participants showed similar and significant improvements from baseline in fasting glucose (LC −17 ± 32 mmol/l, *p* < 0.01; LF −30 ± 29 mmol/l, *p* < 0.001) and total cholesterol levels (LC −22 ± 30 mmol/l, *p* < 0.05; LF −30 ± 43 mmol/l, *p* < 0.001), the LC diet group achieved a statistically significant improvement in their diastolic cardiac function from baseline (*p* < 0.05) compared to the LF group, which showed no significant improvement. This effect occurred despite both groups achieving similar weight loss (LC −2.6 ± 3.3 kg, *p* < 0.01; LF −1.6 ± 2.2 kg, *p* < 0.05). Mean systolic blood pressure, fasting and post- meal triglyceride and insulin levels did not markedly change in the LF group, this is however improved significantly on the LC diet (*p* < 0.05). Improvements in these variables were subsequently observed in the crossover group. Lastly, the authors reported a statistically significant improvement in maximal exercise capacity (2 ± 7 watt, *p* < 0.05) and reduced markers of IR [Homeostatic Model Assessment for IR(HOMA-IR)] in the LC (−0.9 ± 2.4, *p* < 0.05) but not the LF group (−0.1 ± 1.4, *p* >0.05). At the end of the study the use of antidiabetic medication in the LC group was reduced by 88%, compared to 12% in the LF group.

It is of note that within groups, the LF diet induced a greater reduction in both fasting and postprandial low-density lipoprotein (LDL) levels than the LC group from baseline (LC −14 ± 29, *p* < 0.05; LF −26 ± 21, *p* < 0.001). Further to this, the LC diet induced significantly less reduction in LDL levels (*p* < 0.001) after only 2 weeks in the cross over group (LF to LC). An assessment of lipoprotein subfraction particle concentration was not undertaken. This analysis could perhaps have given superior insight regarding cardiovascular disease risk in either group (49). Participants also followed a physical training regime, therefore improvements in biomarkers may be partially attributed to exercise. Whilst the small sample size and short time frame of the dietary intervention pose notable limitations of this RCT, the findings support the hypothesis that a LC diet can improve the pathophysiological manifestations of DMCM.

In a subsequent case study of a 57 year old patient diagnosed with obesity, insulin-dependent T2DM and HFpEF who consumed a LC diet and commenced walking for 1 year, Heilmeyer and von Bibra (108) describe an increase in exercise capacity, normalization of blood sugar, HbA1c and lipid levels despite minimal weight loss. Further, the patient was able to cease subcutaneous insulin injections and reported an improvement in dyspnoea. In both studies the authors emphasize that dietary CHO restriction improves the underlying dysregulations such as IR seen in patients with DMCM (107, 108).

## Implications for Clinical Practice and Conclusion

DMCM is a syndrome that shares characteristics of both HF and T2DM. DMCM decreases a patient's life expectancy, profoundly impacts on their QoL and is difficult to treat (13, 109, 110). Whilst pharmacological management strategies for DMCM are trialed, evidence for effective treatment in clinical trials for patients with DMCM is scarce (73). The current nutritional guidelines for patients with heart disease focus mostly on dietary (saturated) fat and salt reduction (2, 111, 112). However, given that LF diets are not primarily designed to alter CHO intake, they are unlikely to address the metabolic changes due to IR seen in DMCM (46, 113, 114). Targeted food modification should go beyond sodium and fluid restriction and needs to be evidence based.

Literature suggests LC diets present a safe and effective tool for the management of various clinical conditions such as T2DM and other metabolic disorders (55). For patients with DMCM, LC diets are able to modulate the harmful postprandial changes (glucotoxicity and lipotoxicity) and their devastating metabolic effects (62).

LC diets also facilitate body water loss. It is therefore conceivable that symptoms of congestion in HF patients may be attenuated by such a diet through the mobilization of glycogen stores, natriuresis directly induced by ketones and downregulation of the renal SGLT2 system (50). Ketones play an important role in fueling the failing heart. Whilst these molecules can be enhanced pharmacologically, well-formulated LC diets may provide a cost effective and safe option for some patients. The increase in energy dense macronutrients such as fat and protein on a LC diet may help underweight HF patients retain both body fat and muscle mass by preventing insulin resistance mediated catabolism (102, 106). Additionally, with their appetite suppressing effect, LC diets provide an effective tool for patients struggling to lose weight with calorie restricted diets (48, 93).

## Conclusion

We report here our hypothesis, that well-formulated LC diets may be a powerful tool for the prevention and progression of HF. We further depict the suggested mechanisms behind these effects. Clinical trials with LC interventions for patients living with DMCM are warranted.

These trials should first establish the therapeutic impact of CHO restriction on the complex syndrome that is DMCM. Second, due to their analogous effect and the risk of EDKA, LC diets cannot be prescribed for patients taking SGLT2i but could potentially replace them. Carefully designed clinical HF trials, with drug and diet comparison groups are needed to further our understanding of the LC diet's effect on DMCM pathophysiology. Evidence of these trials may allow for the prescription of a LC lifestyle to HF patients who cannot or would prefer not to take SGLT2i medications.

## Data Availability Statement

The original contributions presented in the study are included in the article/supplementary material, further inquiries can be directed to the corresponding author/s.

## Author Contributions

SK-M: conceptualization, original draft preparation, literature search, acquisition, analysis and interpretation of data, and original draft preparation. BR, AO, CZ, and AD supervision and review and editing all stages of the manuscript. All authors have read and agreed to the published version of this manuscript.

## Conflict of Interest

The authors declare that the research was conducted in the absence of any commercial or financial relationships that could be construed as a potential conflict of interest.

## Publisher's Note

All claims expressed in this article are solely those of the authors and do not necessarily represent those of their affiliated organizations, or those of the publisher, the editors and the reviewers. Any product that may be evaluated in this article, or claim that may be made by its manufacturer, is not guaranteed or endorsed by the publisher.
